# Single‐cell RNA‐seq in diabetic foot ulcer wound healing

**DOI:** 10.1111/wrr.13218

**Published:** 2024-09-12

**Authors:** Yan Dong, Mengting Wang, Qianqian Wang, Xiaoliang Cao, Peng Chen, Zhenhua Gong

**Affiliations:** ^1^ Medical School Nantong University Nantong China; ^2^ Department of Burn and Plastic Surgery Affiliated Hospital 2 of Nantong University, The First People's Hospital of Nantong Nantong China; ^3^ Nantong Clinical Medical College Kangda College of Nanjing Medical University Nantong China

**Keywords:** DFU, single‐cell RNA‐seq, therapeutic target, wound healing

## Abstract

Diabetic foot ulcer (DFU) is a chronic and serious complication of diabetes mellitus. It is mainly caused by hyperglycaemia, diabetic peripheral vasculopathy and diabetic peripheral neuropathy. These conditions result in ulceration of foot tissues and chronic wounds. If left untreated, DFU can lead to amputation or even endanger the patient's life. Single‐cell RNA sequencing (scRNA‐seq) is a technique used to identify and characterise transcriptional subpopulations at the single‐cell level. It provides insight into cellular function and the molecular drivers of disease. The objective of this paper is to examine the subpopulations, genes and molecules of cells associated with chronic wounds of diabetic foot by using scRNA‐seq. The paper aims to explore the wound‐healing mechanism of DFU from three aspects: inflammation, angiogenesis and extracellular matrix remodelling. The goal is to gain a better understanding of the mechanism of DFU wound healing and identify possible DFU therapeutic targets, providing new insights for the application of DFU personalised therapy.

## 1 INTRODUCTION

Since the 21st century, with the rapid improvement of people's living standards, the incidence of diabetes is also increasing and gradually becoming one of the major factors threatening people's health. One out of 11 adults in the world has diabetes, and 90% of them have Type 2 diabetes. Diabetes that is not controlled in time can have many negative effects on the structure and function of many organs and systems in the body. Complications of diabetes are traditionally divided into vascular complications and peripheral nerve complications, which mainly affect the heart, kidneys, retina and nerves.^1^ Diabetic foot ulcer (DFU), a serious complication of chronic diabetes, is an infection, ulceration or deep tissue destruction of the foot caused by distal limb neuropathy and vascular disease in diabetic patients.^2^ It can lead to amputation or even life‐threatening complications.

The occurrence of DFU is caused by a combination of multiple factors. Diabetes‐related peripheral neuropathy can damage the distal nerves of the extremities, resulting in decreased pain and progressive numbness, so that even minor trauma can cause the onset and progression of DFU. Vascular complications associated with peripheral arterial disease in diabetes can lead to perfusion defects that reduce blood flow to infected tissue, which in turn can lead to exacerbation of infected ulcers.^3^ In addition, the innate immune response that normally protects against infection is compromised by the acute hyperglycaemia of diabetes, which in turn leads to a severe inflammatory response at the ulcers. Chemokine deficiency, abnormal inflammatory response, vascular and epithelial dysplasia, and fibroblast dysfunction also lead to the progression of DFUs. From this point of view, early diagnosis and early treatment of DFU is very necessary.

The development of systems biology techniques, such as Genomics, Proteomics, Metabolomics and Microbiomics, has been applied to the study of DFU to better understand the disease. The progression of DFU can be predicted from targets and molecules associated with the pathogenesis of DFU.^4^ Single‐cell RNA sequencing (scRNA‐seq) is a study that can discover new cell types, explore dynamic changes and identify specific genes involved in disease progression. scRNA‐seq can be used to detect the RNA expression of individual cells, thereby understanding the changes of each cell in diseases and helping to infer cell function.^5^ In the past few years, research on scRNA‐seq technology has significantly increased, quantifying the gene expression profile of specific cell populations at the single‐cell level, helping us deeply understand the pathogenesis of DFU, and providing new ideas and strategic methods for the clinical treatment of DFU.

**FIGURE 1:**
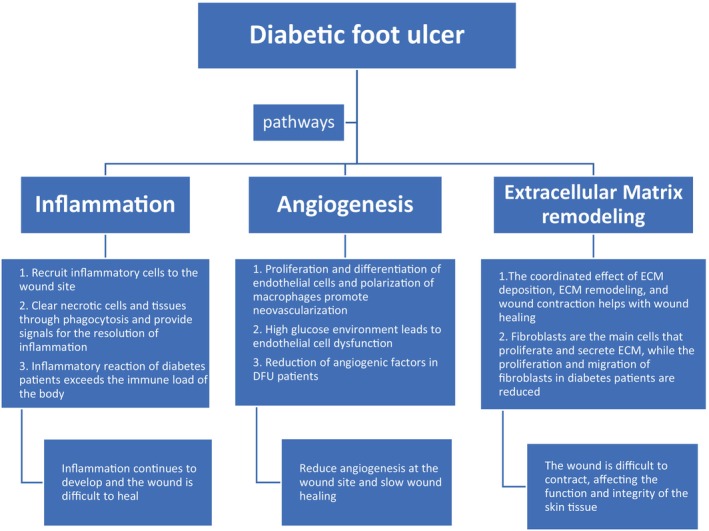
Summarise the various pathways and comorbidities that contribute to the pathology of diabetic foot healing.

This article briefly describes the process of DFU wound healing and the conceptual development and application of scRNA‐seq in wound healing mechanisms. Then, relevant gene targets and possible targeted drugs are searched for, providing new ideas for the clinical treatment of DFU wounds. Finally, it is discussed and suggested that scRNA‐seq will help to elucidate the pathogenesis and pathophysiological changes of DFU and provide appropriate personalised treatment options (Figure 1).

## 2 THE MAIN PROCESS OF WOUND HEALING

The processes of wound healing involve: inflammation, angiogenesis and extracellular matrix (ECM) remodelling. Normal wound healing is a complex biological process that requires the synergistic action of multiple cells, cytokines and the ECM.^6^ Any problem in any of these steps will lead to slow or even non‐healing of the wound, resulting in serious consequences (Table 1).

**TABLE 1:** We identify three processes of DFU wound healing: Inflammation, angiogenesis and extracellular matrix (ECM) remodelling, and summary all findings on DFU scRNA‐seq from three aspects: Cell types, targets, and pathways.

Healing process	Cell types	Target	Pathways
Inflammation	Macrophages	*NFE2L2* *REL* *ETV6* *MAF* *NF1B* ^41^ *FOSB* *JUN* *JUND TNFAIP3* *IL‐1B* *TSC22D3 PIK3IP1* ^46^ *CHI3L1* *TNFAIP 6* *HIF 1A* ^44^	AMPK signalling pathway, IL‐17 signalling pathway, collecting duct acid secretion; ether lipid metabolism, mineral absorption, complement and coagulation cascades^46^ IL‐6, IL‐8, CD28 signalling, iCOS‐iCOSL pathways, RhoGDI and EIF2 signalling; IL6, HIF1A, and ILK signalling^44^
	Neutrophils		
	T cells, B cells, NK cells, monocytes		
	Fibroblasts		
Angiogenesis	Vascular endothelial cells	*RAB17* ^50^ *CCND 1* *ENO 1* *HIF 1a* *MMP 2* *SERPINE1* ^49^ *SLCO2A1* *CYP1A1* ^43^ *ANPEP* *BID* *CYBA* *CYBB* *FCER1G ITGA1* *PLAUR* ^2^	MAPK/ERK signalling pathway^50^ AGE‐RAGE signalling pathway, IL‐17 signalling pathway, Focal adhesion, PI3K‐Akt signalling pathway, ECM–receptor interaction^49^ CCL2‐ACKR1 axis; CCL, VEGF, CALCR, and EDN signalling pathways; IL‐1, IL‐16, LIGHT, CHEMERIN, and IGF signalling pathways; CD70, CSF, BAG, ncWNT, and ANNEXIN signalling pathways; CCL, PROS, EDN, PERIOSTIN, and PARs signalling pathways; FGF, SEMA 3, MK, PIN, and TGFβ signalling pathways^53^
	Multipotential stem cells, Haematopoietic stem cells		
ECM remodelling	Fibroblasts	*MMP 1* *MMP 3* *MMP 11* ^44^ *CENPF* *PTTG 1* *MKI 67* *TOP2 A* ^44^	ECM–receptor interaction^53^
	SMCs		

Abbreviations: DFU, diabetic foot ulcer; scRNA‐seq, single‐cell RNA sequencing; SMC, smooth muscle cell.

### 2.1 Inflammation

Chronic and persistent inflammation is one of the reasons why most chronic wounds are difficult to heal.^7^ The inflammatory phase of wound healing leads to the presence of various inflammatory cells at the site of injury, such as neutrophils, monocytes and pro‐inflammatory macrophages. In the early stages of wound injury, neutrophils are recruited first, attracted by chemical attractants and bacterial endotoxins, and recruited from damaged blood vessels into the wound.^8^ Upon arrival at the wound, neutrophils clear necrotic tissue and pathogens through phagocytosis and the release of reactive oxygen species (ROS), antimicrobial peptides, arachidonic acids and proteolytic enzymes.^9^ Monocytes enter the wound tissue and differentiate into macrophages under the influence of the local environment. Macrophages provide a signal of inflammation resolution to the body by engulfing apoptotic neutrophils. However, when the sustained inflammatory response exceeds the body's immune load capacity, it will cause the body and wound's inflammatory response to continue to develop, ultimately leading to difficulty in wound healing.^10^ In short, in the early stages, macrophages promote inflammation and remove pathogens, wound debris and apoptotic cells.^11^

### 2.2 Angiogenesis

After inflammation is controlled, skin cells and endothelial cells continue to proliferate and differentiate, which can lead to angiogenesis. Angiogenesis not only requires endothelial cell proliferation, migration and differentiation,^12^ but also includes growth promoting or survival factors, proteolytic enzymes, activators of various differentiation and progenitor cell types and suitable microenvironment.^13^ Vascular endothelial cells are crucial cells for angiogenesis and are crucial for wound healing. They can promote angiogenesis and maintain oxygen and nutrient supply at the wound site.^14^ Studies have shown that endothelial cells chronically exposed to high blood glucose become dysfunctional in patients with diabetes, leading to loss of integrity and increasing apoptosis.^15^ It has also been shown that macrophage polarisation has an effect on wound healing, scar formation and angiogenesis.^16^ In the later stage of the repair process, macrophages can inhibit inflammation and secrete factors that regulate the proliferation, differentiation and migration of keratinocytes, fibroblasts and endothelial cells, leading to neovascularisation and wound closure.^11^ Angiogenetic growth factors play an important role in promoting angiogenesis. Angiogenic growth factors play an important role in promoting angiogenesis. In patients with diabetes, angiogenic growth factors (such as vascular endothelial growth factor [VEGF], epidermal growth factor [EGF] and hypoxia‐inducible factor [HIF‐1α]) are reduced, resulting in slow wound healing.^17^ Angiogenic growth factor promotes chronic wound closure in hypoxic and vascularised wounds. VEGF is an angiogenesis‐stimulating protein that enhances diabetic wound healing by inducing endothelial cell migration, increasing vascular permeability and promoting epithelial formation and collagen deposition in wound healing.^18^ HIF‐1α is a transcriptional complex that responds to hypoxia and regulates several target genes, including VEGF. Ischemia and hypoxia in wounds activate HIF‐1α, which up‐regulates the expression of VEGF and its receptor, and thus promotes the angiogenic response, which leads to improvement of tissue ischemia and hypoxia.^19^ In contrast, a high‐glucose internal environment inhibits the protein expression and transcriptional function of HIF‐1α and reduces tissue and cellular responses to hypoxia, thereby inhibiting wound healing.^20^ EGF is produced by platelets, macrophages and monocytes, and its role is to stimulate the growth of epithelial cells, fibroblasts and smooth muscle cells (SMCs) on wounds and to promote chronic wound growth.^21^

### 2.3 ECM remodelling

Skin tissue helps heal wounds through the coordinated effect of ECM deposition, ECM remodelling and wound contraction.^22^ ECM remodelling occurs throughout the whole injury response, beginning with fibrin deposition and ending with collagen‐rich scar formation. Fibroblasts are the major cell type responsible for ECM remodelling in wound healing. Fibroblasts proliferate and secrete ECM components that promote the contraction of the wound bed and the production of collagen, and also remodel the ECM by releasing MMP.^23^ It can provide supporting structures for cell proliferation and migration, and can be responsible for wound contraction to restore skin tissue function and integrity.^24^ However, in DFU, the proliferation and migration of skin fibroblasts decrease, collagen fibres degenerate and break, and the proportion of Type I/III collagen decreases, making it difficult for the wound to heal.^25^

## 3 SINGLE‐CELL RNA SEQUENCING

scRNA‐seq, a new technology that can be used to explore molecular changes in complex cell clusters at the single‐cell level. It is capable of analysing multiple molecular signatures of a single cell,^26^ comprehensively measuring gene expression levels in cells, studying transcriptomic gene variation, revealing new cell types and gaining insight into the molecular drivers of cell function and disease.^27^ scRNA‐seq can provide a viable view of the different stages of differentiation and activation states that are rarely synchronised between cells.^28^ It can facilitate our research into the pathogenesis of diseases and help provide new diagnostic and therapeutic approaches.

The process of scRNA‐seq includes: sample preparation, single‐cell capture, reverse transcription and amplification, library preparation, sequencing and analysis.^29^ One of the key applications of scRNA‐seq is the identification of cell subpopulations based on cell clustering or classification.^30^ The obtained tissue is sequenced at the single cell level and a gene expression matrix is generated for each cell, which is then subjected to dimensionality reduction analysis to identify specific cell subpopulations and clusters. scRNA‐seq can help identify different cell populations, reveal informative cell characteristics, and elucidate key intercellular relationships, allowing for the characterisation of gene expression in specific cell types. By comparing the proportions of different cell types in DFU tissues and control tissues, the most important special cell populations in the ulcer microenvironment of DFU patients could be found, cell heterogeneity could be assessed, possible drug targets could be identified, microenvironment in the affected area of DFU could be improved. In conclusion, scRNA‐seq reveals cell heterogeneity and monitors the progression of DFU disease development, thereby preventing further cell deterioration. In addition, analysis of DFU tissue cells can be used to classify immune cells and their immune mechanisms, and to develop effective clinically targeted therapies in combination with immunotherapy.^31^ Currently, scRNA‐seq has been widely studied in cell population identification, gene diagnosis and therapeutic targets for cancer,^32^ but the research on scRNA‐seq in DFU wound healing has not been fully explored yet. However, it is certain that the use of scRNA‐seq in the diagnosis and treatment of DFU will become an important direction of future research.

## 4 DISCOVERIES AND APPLICATIONS OF DFU HEALING MECHANISMS UNDER scRNA‐SEQ

DFU wounds that do not heal within 12 weeks are considered non‐healing or chronic wounds.^33^ Common features of non‐healing wounds include exudates, recurrent infections, tissue necrosis, defective re‐epithelialisation, reduced angiogenesis and excessive ROS production.^34^ Hyperglycaemia is one of the microenvironmental features of DFU. Excessive glucose inhibits the expression of antioxidants and promotes oxidative stress in the body, thereby exacerbating the inflammatory response.^35^ During the inflammatory phase, hyperglycaemia inhibits the expression of chemokines, which inhibits the recruitment of neutrophils and prevents monocytes and macrophages from entering the wound for phagocytosis, resulting in increased wound oxidative stress. DFU wound is in a chronic hypoxic environment, and oxidative stress affects endothelial cells, keratinocytes and fibroblasts, resulting in dysfunction of endothelial cells, abnormal migration and proliferation of keratinocytes, and impaired proliferation, migration and differentiation of fibroblasts.^36^ Therefore, it also has a major impact on wound vascularisation and ECM remodelling. Delayed wound healing in diabetes is characterised by the following: the increase of MMPs and inflammatory factors (e.g., *TNF‐α*), and the increase of protein degradation.^37^ These will exacerbate tissue necrosis and result in failed wound healing. All of the above changes in the DFU microenvironment will lead to non‐healing of DFU wounds, which will bring great difficulties for subsequent treatment.

### 4.1 Inflammation

Studies have shown that neutrophils and macrophages are capable of releasing large amounts of pro‐inflammatory cytokines, and increased infiltration of pro‐inflammatory cells can lead to delayed healing of chronic ulcers.^38^ Macrophages are mainly derived from haematopoietic stem cells and belong to the monocyte population of immune cells, which are divided into M1 and M2 macrophages. M1 macrophages, classically activated macrophages, are mainly activated by lipopolysaccharides and IFN‐γ and have a pro‐inflammatory effect. M2 macrophages, selectively activated macrophages, are mainly activated by IL‐4 and IL‐13 and have an anti‐inflammatory effect.^39^ Normally, in the early stage of inflammation, M1 macrophages are activated to secrete inflammatory factors to accelerate the development of inflammation. And in the late stage of inflammation, M2 macrophages are activated to promote cell proliferation and tissue remodelling. So the polarisation of macrophages from M1 to M2 phenotype can help to heal wounds.^40^ Li et al. conducted bioinformatics and high‐throughput sequencing data analysis on the GSE 165816 data set, and found that the proportion of M1 and M2 macrophages was higher than that of other cell subpopulations, and the proportion of M1 macrophage clusters was the highest in the non‐healing group of DFU. Then, the enrichment analysis of differentially expressed IRGs (immune‐associated genes, which are essential for immunological infiltration) in M1 macrophages showed that IRGs may be involved in the immune function of DFU, and the immune‐related factors are closely related to the changes in DFU microenvironment.^41^

The immune system can help regulate inflammation and maintain a stable internal environment, but diabetics have a persistent pro‐inflammatory environment and regulated aberrant immune cell expression.^42^ In DFU, the upstream regulatory factors IL‐13 and IFN‐γ are inhibited, and the biological processes such as cell motility of monocytes, migration of dendritic cells and chemotaxis of antigen‐presenting cells are disrupted, resulting in impaired migration profile of immune cells.^43^ Theocharidis et al. analysed the peripheral blood mononuclear cell (PBMC) and found that CCR7^+^CD8^+^ T lymphocytes were highly expressed in DFU‐healed individuals, while NKT cells were highly expressed in DFU‐unhealed individuals. These results suggest that T lymphocytes are associated with wound healing. Pathway analysis of DEG of these T/NKT cells showed that at the systemic level, key immune and inflammatory pathways were suppressed in DFU healers, including IL‐6, IL‐8, CD 28 signalling and iCOS‐iCOSL pathways in T‐helper cells, and activated RhoGDI and EIF 2 signalling. Systems biology analyses revealed suppression of upstream regulators (including *CD 44*, *TGFB 1*, *CCL 5* and *NFKBIA*) in T cells and enrichment of naive T cells in PBMC from DFU healers, which contributed to suppression of immunity and inflammation at the systemic level. These conclusions indicate that the healing of DFU wounds requires a locally activated inflammatory response to progress to the next stage of wound healing, and inhibition of inflammation is required at the systemic level to aid in wound healing.^44^ Based on the important role of immune cells in the occurrence and progression of DFU, Gan et al. analysed the cell abundance of DM, DFU healing, and DFU non‐healing, and found that immune cells (activated CD8^+^ T cells, central memory CD8^+^ T cells, T follicular helper cells, myeloid suppressor cells, NKT cells and monocytes) were highly infiltrated in patients with non‐healing DFU. However, no significant differences were found between patients with and without ulcers.^45^ The results suggest that immune cells may be involved in the healing process of ulcers, but not in ulcerogenesis. Cheng et al. found through scRNA‐seq analysis that the proportion of CD8^+^ T cells was lower in patients with non‐healing DFU than in patients with healing DFU, and the proportion of CD8^+^ T cells was significantly increased in patients with healing DFU; the proportion of B cells and NK cells was also increased in patients with healing DFU. This suggests that the expression of immune cells may play an important role in the process of ulcer healing. Weighted correlation network analysis of all DEGs in GSE 134431 revealed that genes associated with processes such as skin development, epithelial cell differentiation and keratinocyte differentiation, and the IL‐17 signalling pathway were more active in DFU. Genes related to elastic fibre assembly, cell adhesion, biological adhesion, and complement and coagulation cascade are highly expressed in wound healing.^46^ Wound healing is related to proliferation of fibroblasts, endothelial cells and keratinocytes in an oxygen‐rich environment.^47^ However, the proliferation of these cells mentioned above is severely impaired in DFU, and the expression of inflammatory immune cells is increased, leading to prolonged DFU inflammation and delayed wound healing. The role of many immune cells in the healing process of DFU has not been verified, which also plays a guiding role in the exploration of the pathophysiology of DFU.

### 4.2 Angiogenesis

Promoting vascular regeneration in wounds is a key step in wound healing, and endothelial cells are key players. Endothelial cells not only form the inner layer of arteries, veins and capillaries, but also act as endocrine cells mediating immune and inflammatory responses.^48^ Lu et al. conducted scRNA‐seq analysis using data set GSE 165816 and found that vascular endothelial cells were significantly up‐regulated in pathways interacting with the AGE‐RAGE signalling pathway, the IL‐17 signalling pathway, focal adhesion, the PI3K‐Akt signalling pathway and the ECM–receptor interaction conduction pathway. Suppression of the relevant pathways in the vascular endothelium of non‐healing of DFU would all affect DFU angiogenesis and wound healing.^49^ Du et al. found that DFU exhibited a slightly higher proportion of human normal microvascular endothelial cells (HDMECs) compared to healthy controls, but exhibited significantly impaired angiogenesis ability.^50^ It is speculated that the impaired angiogenesis of DFU may be due to a decrease in angiogenesis function, rather than a decrease in cell count. This also provides ideas for further investigation of relevant genes involved in impaired angiogenesis in DFU‐HDMECs. Data based on scRNA‐seq of endothelial cells showed higher levels of autophagy expression in well‐healed wounds. Autophagy is an intracellular degradation process that prevents the accumulation of toxic proteins, removes damaged organelles, maintains cellular homeostasis, and acts as a regulator throughout the stages of wound healing.^51^ Lin et al. compared the scRNA‐seq data of DFU healing and non‐healing states, then found the analysis that autophagy and Sirt 1 signal activation have higher expression levels in diabetes wound healing, playing a crucial role in reducing inflammation and oxidative stress, and promoting wound healing.^52^

In comparing the levels of single‐cell distribution, normal and DFU patients were essentially separated, whereas patients with DM were dispersed between the two. It can be seen that the microenvironment of DFU is more different compared to normal and that there is a gradual change from normal to DFU in patients with DM. Li et al. analysed the GSE165816 data set and found that the number of pluripotent stem cells in DFU was significantly reduced compared to DM, while the concentration of macrophages and haematopoietic stem cells in DFU was significantly increased compared to DM. Subsequent analysis using the GSE199939 data set showed that at the single‐cell level, the stem cell index of pluripotent stem cells and haematopoietic stem cells was significantly lower in DFU than in DM. Although there are a large number of haematopoietic stem cells in DFU, their activity may be very low, which seriously affects the healing of ulcers, so using stem cell agonists may become a method for treating DFU. It is hypothesised that two possible factors in the development of DFU are inflammation and cellular stemness, and that monocytes, haematopoietic stem cells, and pluripotent stem cells are the main distinct cell populations in the ulcer microenvironment of patients with DM and DFU, and that alterations in the ulcer microenvironment may be largely dependent on the marked differences in the number and activity of specific cells.^2^ Wang et al. found that tissue stem cells were closely associated with wound healing, and that tissue stem cells promoted wound healing mainly through ECM–receptor interactions, the PI3K‐AKT signalling pathway and platelet activation. Analysis of the GSE 165816 data set revealed that the expression of *CCL2* was increased in DFU healers compared to DFU non‐healers, along with a significant increase in the number of vascular endothelial cells; *ACKR 1* was enriched at endothelial cell junctions. It is concluded that the CCL2‐ACKR 1 signalling pathway activity of tissue stem cells influences the bioactivity of endothelial cells and ultimately promotes DFU wound healing.^53^ Previous studies have shown that adipose‐derived stem cells can promote wound epithelium formation and angiogenesis, which is consistent with scRNA‐seq results.^54^ In addition, Theocharidis et al. found that a group of SMCs overexpressing the proliferative markers *CENPF*, *PTTG 1*, *MK167*, and *TOP2A* were significantly enriched in DFU healers, suggesting that there are highly proliferative clusters of SMCs in healing DFUs.^44^ Promoting the proliferation and differentiation of SMCs may contribute to wound healing. However, the study of SMCs in DFU from the perspective of scRNA‐seq is still relatively scarce, and its further mechanisms deserve deeper investigation.

### 4.3 ECM remodelling

Studies have shown that wound healing disorders in diabetic patients are closely related to fibroblast differentiation dysfunction, impaired myofibroblast activity and insufficient ECM production.^55^ Theocharidis et al. found through analysis of scRNA‐seq results that fibroblasts exhibited significant heterogeneity. The fibroblast population overexpressed in DFU‐healed patients is designated HE‐Fibro, and in‐depth analysis of its subpopulation reveals that high expression of genes related to ECM remodelling (*MMP1*, *MMP3*) and immunity/inflammation (*CHI3L1*, *TNFAIP6*) is conducive to ulcer wound healing. During DFU healing, multiple immune and inflammatory pathways are activated in fibroblasts, including *IL‐6*, *HIF‐1α* and *ILK* signalling. In addition, upstream regulatory genes such as *TNF*, *HIF‐1α* and *IL‐6* are activated, all of which promote wound healing. The enrichment analysis of HE‐Fibro and M1 macrophages showed that they were significantly enriched in the healing inflammatory and early proliferative stages. However, in the later stage of wound healing, there was no significant enrichment of the two. Therefore, the enrichment of HE‐Fibro and M1 macrophages in the initial stage is essential for wound healing. It is noteworthy that HE‐Fibro has a specific spatial distribution and is mainly enriched in the wound bed to promote wound healing. The wound bed shows higher expression of HE‐Fibro‐related genes such as *IL‐6*, *TNFAIP 6*, *MMP1* and *CHI3L1*. The analysis of the most enriched gene, *CHI3L1*, reveals that HE‐Fibro had stronger adhesion ability and lower migration ability, and could anchor on ECM and mediate ulcer healing through secreting molecules.^44^

## 5 THERAPEUTIC TARGETS OF DFU UNDER scRNA‐SEQ

Counteracting the immune and inflammatory response is a major focus of DFU therapy. It has been mentioned above that M1 macrophage clusters play an important role in DFU healing. Li et al. compared the different IRGs of scRNA‐seq and bulk RNA‐seq of M1 macrophages and constructed a protein–protein interaction network, and found that *NFE2L2*, *REL*, *ETV6*, *MAF* and *NF1B* may play key roles in the transcriptional regulation of the IRGs as the central genes.^41^ The experiment verified that the expression of *NFE2L2*, *REL*, *ETV6*, *MAF* and *NF1B* was higher in the blood of DFU patients. This suggests that targeting the above transcription factors can help modulate immune‐related factors as a way to improve the dysregulated microenvironment of DFU. CD8^+^ T cells are important immune cells and are crucial in combating infections.^56^ Cheng et al. analysed the data of CD8^+^ T cells and found that the target genes *FOSB*, *JUN*, *JUND* and *TNFAIP3* were highly expressed in DFU, while the two genes *TSC22D3* and *PIK3IP1* were low expressed in DFU. The first three target genes are involved in the IL‐17 signalling pathway, which mediates the inflammatory response and leads to delayed wound healing.^57^ The IL‐17 family consists of a group of pro‐inflammatory cytokines that play an active role in host defence but have been implicated in the pathogenesis of a variety of immune‐mediated diseases. IL‐17 family signalling can lead to prolonged inflammation and delayed wound healing.^58^ Experiments have shown that the IL‐17 family can exhibit synergistic effects with other pro‐inflammatory cytokines (e.g., TNF‐α, IL‐1 and IL‐6),^59^, ^60^ then affect the repair of DFU tissue. The latter two target genes may have an impact on the function of CD8^+^ T cells in DFU with anti‐inflammatory and immunosuppressive effects.^46^ Gan et al. found through correlation analysis between DEG and important immune‐related genes that IL‐1B is highly expressed in patients with DFU healing.^45^ IL‐1B is a pro‐inflammatory factor that plays a crucial role in the immune pro‐inflammatory process.^61^ Therefore, IL‐1B may affect the DFU healing process by regulating inflammation. In summary, these target genes may play a role in the regression of subsequent DFU inflammation and wound healing, which is a possible important direction for our follow‐up research. Indole‐carboxylamide type mast cell stabiliser MCS‐01 is an effective inhibitor of mast cell degranulation. Tellechea et al. analysed scRNA‐seq results and found that samples treated with MCS‐01 influenced immune cells to promote wound healing by affecting acute inflammation‐related genes such as NF‐κB and STAT 3.^62^ MCS‐01 primarily alters gene expression in mast cells, monocytes and keratinocytes. It can promote wound healing by activating inflammatory pathways and transforming low‐grade chronic inflammation into an acute inflammatory response. In conclusion, local mast cell stabilisation is a potential treatment for DFUs.

In recent years, therapies targeting angiogenesis have become a popular strategy for treating diabetic wounds.^63^ Therefore, inhibition of endothelial cell apoptosis, improvement of endothelial cell homeostasis, and promotion of angiogenesis are a key focus in the treatment of DFU. Du et al. showed through single‐cell and bulk sequencing analyses that the *RAB 17* gene was significantly down‐regulated in HDMEC in DFU compared with the healthy group.^50^ Through studying the expression of HIF‐1 α and VEGF‐A, as well as the length and number of blood vessels, it was found that overexpression of *RAB 17* could enhance angiogenesis. Functional enrichment analysis showed that *RAB 17* mediated the activation of the MAPK/ERK signalling pathway and promoted angiogenesis in HDMECs. This shows a positive correlation between *RAB 17* gene expression and angiogenesis. Lu et al. found in their research that *CCND 1*, *ENO 1*, *HIF 1a*, *MMP 2* and *SERPINE 1* are significantly reduced in DFU non‐healers, are co‐expressed with CD 31 in the vessel wall, and that their absence impedes angiogenesis.^49^ In an analysis of DFU healing and non‐healing signalling pathways, *CCL*, *VEGF*, *CALCR* and EDN were found to be most active in the functional activity of endothelial cells. *IL‐1*, *IL‐16*, *LIGHT*, *CHEMERIN* and IGF were expressed only in DFU non‐healing wound tissue; however, *CD 70*, *CSF*, *BAG*, *ncWNT* and *ANNEXIN*, on the other hand, were expressed only in DFU healing tissues. Cytokines such as *CCL*, *PROS*, *EDN*, *PERIOSTIN* and PAR were more highly expressed in DFU healing tissues than in DFU non‐healing tissues; while cytokines such as *FGF*, *SEMA 3*, *MK*, *PIN* and *TGF‐β* were more highly expressed in DFU non‐healing tissues than in DFU healing tissues.^53^ Although the mechanism of the genes obtained from the above scRNA‐seq in diabetes angiogenesis is still unclear, it can be determined that these targets may be used as therapeutic targets to promote angiogenesis and diabetes wound healing in the future. Oxidative stress is one of the reasons for the slow healing of wounds in DFU, so the protection of endothelial cells from oxidative stress‐induced injury may be a promising therapeutic target for accelerated cutaneous wound healing.^64^ Theocharidis et al. found that *SLCO2A1* and *CYP1A1* gene expression was up‐regulated in DFU non‐healers, which were mainly expressed by clusters of vascular endothelial cells, and they were only expressed in DFU, suggesting that these genes might be investigated as target genes specific to DFU therapy.^43^ What is more, IL‐13 and INF‐γ were also suppressed in DFU, so activation of both might help promote DFU healing.

Stem cells are critical to the wound‐healing process. Stem cells act as key ‘seed cells’ that recruit macrophages and endothelial lineage cells into ischemic and injured tissues, where they secrete growth factors and establish a favourable microenvironment for wound repair.^65^ Based on three cell types: monocytes, haematopoietic stem cells and pluripotent stem cells, seven target genes were screened for significant overexpression in DFU: *ANPEP*, *BID*, *CYBA*, *CYBB*, *FCER1G*, *ITGA1* and *PLAUR*, and six drugs with potential for the treatment of DFU were identified by pharmacogenetic network analysis: CYCLOSPORINE, SIMVASTATIN, CURCUMIN, LUTEOLIN, APIGENIN and CHRYSIN.^2^ These target genes and possible drugs offer new directions for the treatment of DFU wounds. Some studies have investigated the above‐targeted drugs and found that Chrysin (LCR) is a biologically active compound found in plants with anti‐inflammatory and antioxidant activities and also enhances autophagy.^66^ As mentioned above, Sirt1 is closely related to the cell viability and apoptosis level of HUVECs, and it was found that LCR could promote autophagy and attenuate oxidative stress‐induced apoptosis in HUVECs by increasing the expression and enhancing the activity of autophagy‐associated protein Sirt1, so LCR was considered as a possible targeting agent for the treatment of DFU.^52^

Theocharidis et al. found through analysis of scRNA‐seq results that specific subtypes of fibroblasts are key factors in the healing of DFU, and targeted therapy may be a feasible treatment method. scRNA‐seq of HE‐Fibro in wound healing of DFU patients showed that fibroblasts overexpressing *MMP 1*, *MMP 3*, *MMP 11*, *HIF 1A*, *CHI3L1* and *TNFAIP 6* genes were able to promote wound healing and that HE‐Fibro was able to preferentially localise toward the wound bed. Therefore, future studies can enable fibroblasts to participate in DFU wound healing as an immunomodulatory factor and help to pre‐enrich HE‐Fibro at DFU ulcers.^44^

## 6 CONCLUSION AND PROSPECT

The process of wound healing includes inflammation, angiogenesis and ECM remodelling. In the DFU, however, the microenvironment of hyperglycaemia and chronic hypoxia leads to alterations in all three of these processes, thereby affecting wound healing. During the process of inflammation, immune cells play a major role, including macrophages, neutrophils, T cells, B cells, NK cells and monocytes. scRNA‐seq analysis shows that the expression of the correct immune cells can help ulcer healing, but a disproportionate number of immune cells can lead to delayed wound healing in DFUs. Endothelial cells play a major role in the progress of angiogenesis. Maintaining endothelial cell homeostasis and promoting endothelial cell pathway transmission will all contribute to ulcer healing. In addition, activation of stem cells also helps to alter the proportion of cells in the DFU microenvironment and promotes endothelial cell pathway transmission, which in turn promotes angiogenesis and wound healing. By activating specific signalling pathways, the expression of specific genes and cytokines is regulated, thereby inhibiting endothelial cell apoptosis and promoting angiogenesis and wound healing. During the progress of ECM remodelling, fibroblasts proliferate and secrete ECM components that promote contraction of the wound bed and collagen production, as well as remodel the ECM by releasing MMPs, thereby promoting wound healing. scRNA‐seq results also show that specific subpopulations of fibroblasts could be enriched at the wound site and participate in the immune‐inflammatory response and ECM remodelling process. Therefore, pre‐enrichment of specific subpopulations of fibroblasts in wounds may be a new direction for targeted therapy.

scRNA‐seq provides a comprehensive characterisation of the DFU ecosystem, discovering new cell types and their interactions. scRNA‐seq experiments on current DFU allow the discovery of changes in the internal environment of DFU at the single cell level and the wound healing mechanism can be explored. The subpopulations, genes, molecules and immune regulations of these cells are studied to discover their regulation of DFU wound healing, thus helping to identify targets for DFU therapy.

However, there are still many shortcomings of scRNA‐seq in DFU that need to be addressed. Sample collection of DFU tissues is a major difficulty. Although the number of DFU patients is large, the number of samples that meet the requirements of experimental specimens is scarce because most of the DFU patients are also accompanied by other systemic diseases, which may affect the results of the experiments. In addition, since most of the wound tissues are collected during surgical procedures, where there are many uncontrollable factors and where it is important not to interfere with the patient's postoperative wound repair, so the number of tissues collected that can be used is very limited. The selection of skin tissue from different sites, and the age and gender of the study subjects can also have a significant impact on the results of the study. The preservation of tissue samples during transport from the operating room to the laboratory is another major challenge. Delays in transfer time and inappropriate preservation methods can both lead to a decrease in cell integrity.^67^ It has also been shown that different methods of dissociation can lead to differences in skin cell proportions, ultimately biasing the results of the study.^68^ And the laboratory environment makes it difficult to mimic the complex interactions between cells and the microenvironment in DFU.^69^ Therefore, the problems we face still need to be improved and solved. With the refinement of experimental methods, scRNA‐seq in the diagnosis and treatment of DFU will be a popular direction for future research.

Finally, reviewing the latest applications of scRNA‐seq in DFU, scRNA‐seq can not only reveal the mechanisms of different cell phenotypes related to DFU progression in damaged tissues, but also discover targets through data analysis, thereby better constructing treatment plans. With the increasing application of scRNA‐seq in various problem‐solving, scRNA‐seq will help us gain a more comprehensive understanding of the physiological and pathological mechanisms of DFU and evaluate therapeutic interventions focused on one or more cell types, and may even help us discover new cells. With the deepening of research, it is possible to vertically compare the cellular changes of DFU specimens collected from the same patient at multiple time points of wound healing in the future, in order to find more effective treatment methods.

## AUTHOR CONTRIBUTIONS

Zhenhua Gong conceived and designed the review. Yan Dong and Mengting Wang searched the literature and wrote the review. Qianqian Wang, Xiaoliang Cao and Peng Chen revised the manuscript. All authors have read and approved the final manuscript.

## CONFLICT OF INTEREST STATEMENT

The authors declare no conflicts of interest.

## Data Availability

All included studies and their data sources are cited in the references.
